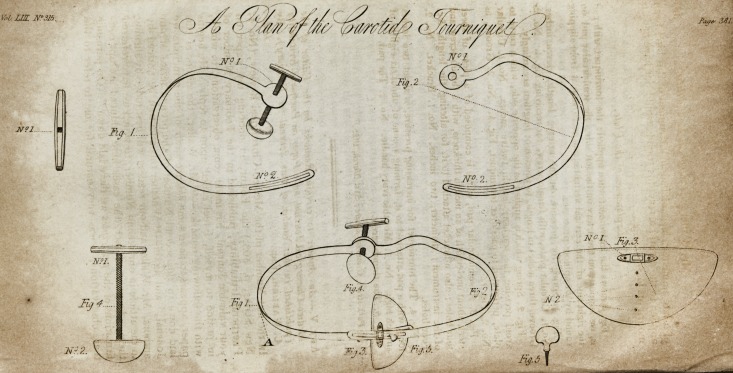# Instrument for Compressing the Carotid Artery

**Published:** 1825-05

**Authors:** C. Powell Blackett

**Affiliations:** Surgeon, &c. &c.


Art. IV. – *Instrument for compressing the Carotid Artery.*
By C. Powell Blackett, Esq. Surgeon, &c. &c.

Sensible that your desire is at all times to do good to mankind, —to improve, by every means, each of the respective branches of surgery, medicine, &c.—to carry a cheering light into the gloomy mansions of sickness and sorrow, by removing, as far as human power is able, the causes of those misfortunes,—and to afford to man ease and quiet, under the various afflictions with which it may please the great Ruler of nature to visit him, I am encouraged to claim your notice, and call your attention to a newly-invented surgical instrument; the plan of which I have the honour of submitting to your consideration.

The most partial and inattentive observer must long since have perceived, with painful regret, the frequent instances of attempts made upon life by the hands of man himself; and his grief must at the same time have been deep and poignant, when he reflected that, on some occasions, life might probably have been preserved, had any means been devised for compressing the arteries, and obviating those effects which have been but too frequently followed. Sensible of this desideratum in the instruments of surgery, my mind has often been employed in the consideration of the best way of supplying its want; and now I beg to offer for your insertion the description of a tourniquet, which I have invented for compressing, in cases of accident or suicide, the carotid artery, when it is either divided, punctured, or lacerated. In cases of gunshot wounds, &c. it will be found equally serviceable and beneficial. It is not from a wish to take to myself any share of praise, that I say, in many instances, for want of an instrument of this kind, death has followed, when life might otherwise have been preserved. In the army, and particularly the navy (in which I have had the honour to serve), this has not unfrequently, I am convinced, been the result. It often happens that, from want of ability and practical knowledge, or even sufficient nerve, the person immediately applied to upon such occasions cannot succeed in securing the artery, and prevent the consequent effusion of blood, attended so commonly with fatal consequences. I beg to observe, that it appears to me this instrument will supply the want so long felt.

Admitting even that the happy effects now anticipated are not completely realised, and that life is not preserved, still in cases, for instance, of attempted self-destruction, the unhappy victim, by the application of this instrument, has time procured to enable him to settle his worldly affairs, and likewise turn his thoughts to higher and better purposes. Without attaching blame to any, I am strongly of opinion that, in the recent case

### A Plan of the Carotid Tourniquet.

of a late eminent nobleman, had an instrument of this kind been applied to the carotid artery, his friends would not have had to mourn his loss.

### *Explanation of the Plate.*

Fig. A shows the instrument when applied: the tourniquet with pad is, we suppose, on the artery on the left side; the semicircular pad on the right side of the cervical vertebræ, so as to stop it from slipping round by the spinous processes.

Fig. 1. Spring for pressure on the artery; length, eleven inches. No. 1. Circular plate, with female screw, for receiving Fig. 4. No. 2. Groove, three inches in length, to shorten or lengthen accordingly.

Fig. 2. Spring which is applied on the opposite side, with a curve to avoid pressing the trachea; length, twelve inches. No. 1. Circular plate, perforated to pass over tourniquet, Fig. 4. No. 2. Groove, as No. 2, Fig. 1.

Fig. 3. A semicircular pad, which is composed of a piece of sheet steel, about one-eighth of an inch in thickness, with a thumb-screw and four holes; also a bracket attached to it, for altering the length, &c. of the instrument; diameter, two inches. No. 1. Bracket. No. 2. Four holes.

Fig. 4. The tourniquet for increasing pressure, is about two inches long, with a pad attached to it, forming a cone of about six-eighths of an inch high. No. 1. Movable ivory handle. No. 2. The pad.

Fig. 5. The thumb-screw.

11, *Park-street, Grosvenor-square; 31st March,* 1825.

## Figures and Tables

**Fig. 1 Fig. 4 Fig. 2 Fig. 3 Fig. 5 f1:**